# Vignette on Canetti, crowds, and the self in times of COVID-19

**DOI:** 10.3325/cmj.2020.61.307

**Published:** 2020-08

**Authors:** Ivana Rosenzweig

**Affiliations:** 1 Sleep and Brain Plasticity Centre, Department of Neuroimaging, Institute of Psychiatry, Psychology and Neuroscience (IoPPN), King's College London, London, UK; 2 Sleep Disorders Centre, Guy's and St Thomas’ NHS Foundation Trust, London, UK *ivana.1.rosenzweig@kcl.ac.uk*

In this extraordinary issue of the *Croatian Medical Journal*, published in the middle of the Coronavirus Disease 2019 (COVID-19) pandemic, we examine its impact across all sections of the wider society. The starting point of our exploration engages with the personal microcosmos of self-discovery during the bizarre and abnormal times of lockdown, beautifully narrated by Calisher (1). Calisher’s self-deprecating, sharp wit and narration are reminiscent of Ephraim Kishon’s (1924-2005) work from the 1960s and 1970s, in which he, as a European émigré and a refugee from the Hungarian Communist regime, describes his life and struggles in the newly founded state of Israel. It reminds us of the importance of humor as a uniquely adaptive defense mechanism that can serve to ward off anxiety, and which can help us to manage conflict; the same humor can provide a pivotal platform to create our new inimitable value system, new meanings, perspectives, and new modes of relating to any imposed and sudden changes in our environment and our lifestyle over which we have little, if any, control (2).

Next, Đogaš et al (3) bring us from the narrow, personal focus, back to wider implications of the societal adjustment in times of pandemic. In the cross-sectional survey on 3027 respondents, Đogaš et al explore lifestyle habits and mood changes in the Croatian general population during the COVID-19 quarantine. The findings are a striking reminder of intrinsic vulnerabilities and gender differences, all still gravely embedded in our society in the 21st century. The authors rightly conclude that aberrant lifestyle behaviors during the quarantine, including significant increases in addictive behaviors such as smoking and drinking, need to be recognized. Perhaps even more importantly, they also need to be addressed in a concerted way, so that appropriate preventative and supervisory measures, including essential public health treatment pathways, can be developed as a matter of urgency. Feasibly, there is a role for humor and its redeemable adaptive qualities here too, but if so, the policy makers would be well served to remember that combining threats and humor in their health messages may be inversely understood, and acted upon, by differential strata of the population (4).

In a different country, but in a somewhat similar vein, Rosenzweig et al (5) reflect on comparable findings of a survey in the United Kingdom, where substantial changes to sleep and anxiety patterns have been reported since the lockdown measures were introduced (6). We are reminded of a growing number of patients who report longer-term physical and psychological effects of COVID-19, some of which may be due to sequelae of a direct central nervous system involvement (7).

We finally reach the end of our journey through the current pandemic times in a thoughtful psychoanalytical work by Okorn et al (8). The authors altruistically allow us to share in their thoughts and experiences of the impact that isolation has played on their patients, many of whose lives have been stamped upon by the previous war and other atrocities. However, the authors allow us to glimpse hope, and they suggest ways in which sublimation and joint/group work may help in constructively forging the way forward.

In conclusion, let me add my own personal flavor of our current times. It is unquestionable that we, as biologic beings whose sense of “self” fires within mysterious brain networks that can be tamed (9), flourish in a crowd. The bigger, the better, the more loud and global, the more powerful, and less alone we feel. I wonder, however, whether by trying to avoid the associated pain and anxiety that the imposed isolation inevitably brings, we may in fact forgo our chance for a transcendental change as individuals, and as a society. In the words of a powerful visionary, Elias Canetti (1905-1994), I see an important echo for our times too:

“THERE IS NOTHING that man fears more than the touch of the unknown… It is only in a crowd that one can become free of this fear of being touched. That is the only situation in which the fear changes into its opposite…He feels him as he feels himself. Suddenly it is as though everything were happening in one and the same body... The crowd particularly likes destroying houses and objects… It is true that the noise of destruction adds to its satisfaction. The noise is a promise of the reinforcements the crowd hopes for, and a happy omen for deeds to come. Sculptures of solid stone have been mutilated beyond recognition…The destruction of representational images is the destruction of a hierarchy which is no longer recognized. It is the violation of generally stablished and universally visible and valid distances. The solidity of the images was the expression of their permanence. They seem to have existed for ever, upright and immovable; never before had it been possible to approach them with hostile intent. Now they are hauled down and broken to pieces. In this act the discharge accomplishes itself…ONE OF THE most striking traits of the inner life of a crowd is the feeling of being persecuted, a peculiar angry sensitiveness and irritability directed against those it has once and forever nominated as enemies. These can behave in any manner, harsh or conciliatory, cold or sympathetic, severe or mild-whatever they do will be interpreted as springing from an unshakable malevolence, a premeditated intention to destroy the crowd, openly or by stealth.

In order to understand this feeling of hostility and persecution it is necessary to start from the basic fact that the crowd, once formed, wants to grow rapidly. It is difficult to exaggerate the power and determination with which it spreads.” Elias Canetti’s commentary of our times (10).
